# Carnivore hotspots in Peninsular Malaysia and their landscape attributes

**DOI:** 10.1371/journal.pone.0194217

**Published:** 2018-04-04

**Authors:** Shyamala Ratnayeke, Frank T. van Manen, Gopalasamy Reuben Clements, Noor Azleen Mohd Kulaimi, Stuart P. Sharp

**Affiliations:** 1 Department of Biological Sciences, Sunway University, Bandar Sunway, Malaysia; 2 U.S. Geological Survey, Northern Rocky Mountain Science Center, Interagency Grizzly Bear Study Team, Bozeman, Montana, United States of America; 3 Rimba, Kuala Lumpur, Malaysia; 4 Ex-Situ Conservation Division, Department of Wildlife and National Parks, Kuala Lumpur, Malaysia; 5 Lancaster Environment Centre, Lancaster University, Lancaster, United Kingdom; National University of Singapore, SINGAPORE

## Abstract

Mammalian carnivores play a vital role in ecosystem functioning. However, they are prone to extinction because of low population densities and growth rates, and high levels of persecution or exploitation. In tropical biodiversity hotspots such as Peninsular Malaysia, rapid conversion of natural habitats threatens the persistence of this vulnerable group of animals. Here, we carried out the first comprehensive literature review on 31 carnivore species reported to occur in Peninsular Malaysia and updated their probable distribution. We georeferenced 375 observations of 28 species of carnivore from 89 unique geographic locations using records spanning 1948 to 2014. Using the Getis-Ord Gi*statistic and weighted survey records by IUCN Red List status, we identified hotspots of species that were of conservation concern and built regression models to identify environmental and anthropogenic landscape factors associated with Getis-Ord Gi* *z* scores. Our analyses identified two carnivore hotspots that were spatially concordant with two of the peninsula’s largest and most contiguous forest complexes, associated with Taman Negara National Park and Royal Belum State Park. A cold spot overlapped with the southwestern region of the Peninsula, reflecting the disappearance of carnivores with higher conservation rankings from increasingly fragmented natural habitats. Getis-Ord Gi* *z* scores were negatively associated with elevation, and positively associated with the proportion of natural land cover and distance from the capital city. Malaysia contains some of the world’s most diverse carnivore assemblages, but recent rates of forest loss are some of the highest in the world. Reducing poaching and maintaining large, contiguous tracts of lowland forests will be crucial, not only for the persistence of threatened carnivores, but for many mammalian species in general.

## Introduction

Few taxonomic groups elicit as much conservation attention as mammalian carnivores [1–3]. Carnivores of various sizes play a crucial role influencing the composition and dynamics of ecological communities [4]. The loss of apex predators has been linked to cascading consequences for smaller herbivores regulated by mid-order predators [5–6], which in turn can influence plant growth and recruitment via altered patterns of herbivory, seed predation, and seed dispersal [3,4,7,8]. Charismatic carnivores often serve as conservation flagships [9], and when their area and resource requirements encompass those of numerous species, they serve as conservation umbrellas [10–12]. Carnivore presence may be linked positively with biodiversity [13,14], habitat integrity [15], and ecological processes [4]. Ironically, the very characteristics that make carnivores such effective conservation surrogates also make them extinction-prone.

Mammalian carnivores are vulnerable to extinction mainly due to habitat loss and human-induced mortality [16,17]. Carnivores in general occupy the higher region of ecological food webs, composing a relatively small fraction of ecological biomass and requiring a healthy prey base to maintain viable populations. Large carnivores need substantial areas that support the prey they subsist on and some level of functional landscape connectivity for persistence. Loss of habitat and prey renders them prone to conflicts with humans [18–21]. Furthermore, carnivores are prime targets for poachers seeking valuable body parts or trophies [22–25] and their life histories often hinder recovery from population declines [26]. Not surprisingly, many carnivore populations across the globe are threatened [27].

Carnivore species richness in Peninsular Malaysia is one of the highest in the world, with 31 species representing seven families recorded to date [28] (Table 1). Sixteen species are listed as critically endangered, endangered, vulnerable, or near threatened at the global level [27]. The most recent local assessment of the conservation status of mammals lists 14 carnivore species as threatened or near threatened in Peninsular Malaysia [28].

**Table 1:** Carnivores of Malaysia with 2015 IUCN conservation status, and Peninsular Malaysia conservation status in 2007 and 2009 based on percent change in area of occupancy and expert opinion [28]. Although 31 species are listed, three species may not be indigenous or extant. The highest threat status, based on IUCN Red List criteria A–E [29] is reported for each species. EX = extinct, CE = critically endangered, EN = endangered, VU = vulnerable, NT = near threatened, LC = least concern.

	Family	Species	Common name	IUCN 2015 Red List status	Peninsular Malaysia 2009 Red List status^a^
1	Canidae	*Cuon alpinus*	Dhole	EN	NT
2	Felidae	*Panthera tigris*	Tiger	CE^b^	EN
3	Felidae	*Panthera pardus*	Leopard	NT	EN
4	Felidae	*Neofelis nebulosa*	Clouded leopard	VU	NT
5	Felidae	*Pardofelis marmorata*	Marbled cat	NT	LC
6	Felidae	*Prionailurus bengalensis*	Leopard cat	LC	LC
7	Felidae	*Prionailurus viverrinus*	Fishing cat^c^	EN	VU
8	Felidae	*Prionailurus planiceps*	Flat-headed cat	EN	NT
9	Felidae	*Catopuma temminckii*	Asian golden cat	NT	LC
10	Herpestidae	*Herpestes javanicus*	Javan mongoose	LC	LC
11	Herpestidae	*Herpestes edwardsii*^b^	Indian gray mongoose^d^	LC	EX
12	Herpestidae	*Herpestes brachyurus*	Short-tailed mongoose	LC	LC
13	Herpestidae	*Herpestes urva*	Crab-eating mongoose	LC	EN
14	Mustelidae	*Martes flavigula*	Yellow-throated marten	LC	NT
15	Mustelidae	*Mustela nudipes*	Malay weasel	LC	NT
16	Mustelidae	*Aonyx cinerea*	Asian small-clawed otter	VU	LC
17	Mustelidae	*Lutra sumatrana*	Hairy-nosed otter	EN	LC
18	Mustelidae	*Lutra lutra*^c^	Eurasian otter^e^	NT	EN
19	Mustelidae	*Lutrogale perspicillata*	Smooth otter	VU	LC
20	Prionodontidae	*Prionodon linsang*	Banded linsang	LC	NT
21	Ursidae	*Helarctos malayanus*	Malayan sun bear	VU	VU
22	Viverridae	*Viverricula indica*	Small Indian civet	LC	NT
23	Viverridae	*Viverra tangalunga*	Malay civet	LC	LC
24	Viverridae	*Viverra megaspila*	Large spotted civet	VU	EN
25	Viverridae	*Viverra zibetha*	Large Indian civet	NT	NT
26	Viverridae	*Cyanogale bennetti*	Otter civet	EN	EN
27	Viverridae	*Paguma larvata*	Masked palm civet	LC	LC
28	Viverridae	*Paradoxurus hermaphroditus*	Common palm civet	LC	LC
29	Viverridae	*Hemigalus derbyanus*	Banded civet	NT	LC
30	Viverridae	*Arctogalidia trivirgata*	Small-toothed palm civet	LC	LC
31	Viverridae	*Arctictis binturong*	Binturong	VU	LC

^a^[28]

^b^IUCN changed status of tiger from endangered to critically endangered in 2015

^c^Evidence for an indigenous population in Peninsular Malaysia is inconclusive [30,31].

^d^Considered introduced with records only from the west coast of the peninsular; no recent records [32].

^e^No proof that the species existed in Peninsular Malaysia [33], but Azlan and Sharma [34] reported a road kill in Terengganu.

Carnivores are difficult to study by direct observation because many are nocturnal and secretive, and exist at intrinsically low population densities [35]. Early surveys in Peninsular Malaysia used traps, direct observation, signs, and road kills to infer species presence. Technological advances such as remote cameras have made it possible for recent surveys to document a greater variety of carnivore species and make inferences about their behavior, habitat use, distribution, and community composition [36–39]. All these techniques have their limitations, but collectively can provide useful information about where a species occurred, its frequency or rarity of occurrence, and its possible vulnerability or adaptability to land use change.

The demand for tropical forest products or land for agriculture continues to exert enormous pressure on natural forests in Peninsular Malaysia. The conversion of tropical rainforest includes small-scale swidden agriculture, rural and urban expansion, and large-scale commercial agriculture [40,41]. A major cause of tropical forest loss has been the conversion of secondary forest to industrial plantations including oil palm and rubber [42–45]. Future changes in land use are inevitable as human populations grow and the country seeks further economic development through commerce in agriculture and timber extraction. Although Southeast Asia has few documented carnivore extinctions as a region [46], local extinctions of multiple forest-dependent species have presumably occurred. Ranges of some species will likely shrink and fragment, predisposing those remaining populations to even greater extinction risk [47]. For example, tigers (*Panthera tigris*), a valuable species to gauge the success of landscape conservation, are experiencing substantial range contraction in Peninsular Malaysia due to high rates of human-induced changes to the landscape and increased poaching pressure [48,49]. However, we know little about the status and ecological requirements of the vast majority of carnivores in Peninsular Malaysia, nor where the most sensitive and diverse carnivore communities are likely to persist.

Here, we identify regions of high priority for carnivore conservation in Peninsular Malaysia and associated landscape factors. Using data on carnivore species distributions from published surveys and records in combination with geographic information systems (GIS) data on landscape variables, we 1) identify priority regions for carnivore conservation and 2) determine associated environmental and anthropogenic landscape gradients.

## Methods

### Study area

Peninsular Malaysia (130,598 km^2^) is located within the Sundaland subregion of tropical East Asia, which includes Borneo, Sumatra, Java, and surrounding islands, including Bali [50]. In December 2015, human population size was over 24 million with population densities (excluding Federal territories) ranging from 40 individuals/km^2^ in Pahang to 1600/km^2^ in Penang [51]. Malaysia’s climate is typical of the tropical Sundaland subregion with abundant rainfall and warm temperatures that fluctuate little throughout the year. The principal vegetation of tropical rainforest dominated by Dipterocarps is floristically the richest of all the world’s forests [46,52]. The nation’s economy is based on minerals, particularly oil and tin, and agricultural produce; rice and food crops are mainly for domestic consumption, but rubber, palm oil, and timber are the principal earners of foreign exchange [52]. Conversion of tropical forest to other forms of land use has been rapid in Malaysia. In a 30-year period, dryland forest declined from 64% of Peninsular Malaysia’s total area to less than 50% by 1990 and swamp forests declined from 14% to 8% [52]. Over a 30-year period (1975–2005), 3.6 Mha of land were converted to oil palm plantations, resulting in a 20% reduction in forest cover [53]. Rubber plantations that yield both latex and timber are rapidly expanding to replace natural forests designated for timber production under sustained yield, and 375,000 ha of monoculture timber are projected to replace natural rainforest habitat by 2020 [44].

### Literature search and data treatment

**Fig 1:**
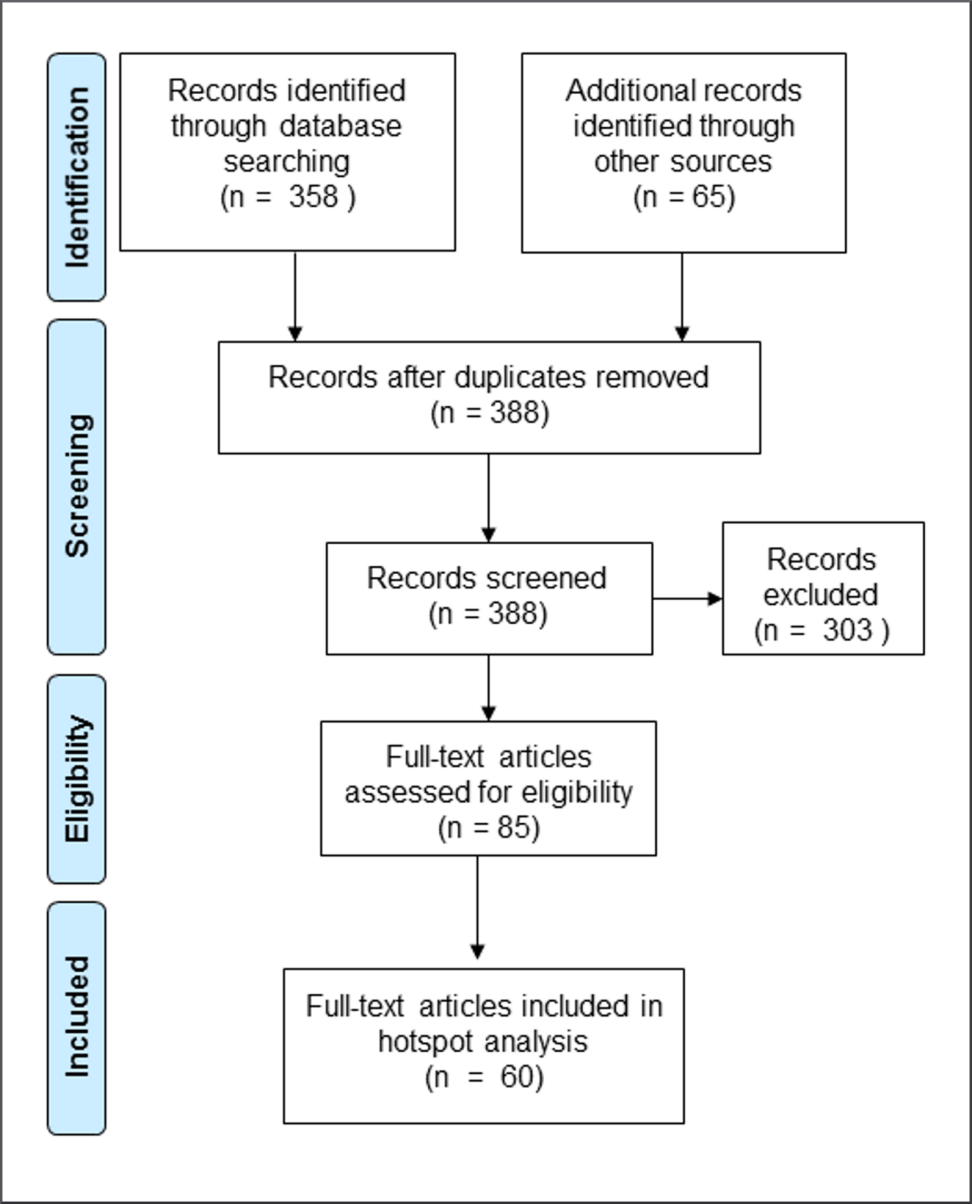
Procedure for the selection of studies of mammalian carnivores in Peninsular Malaysia with records collected during 1948–2014.

We first obtained a species list of carnivores in Peninsular Malaysia [28]. Next, we carried out a literature search for carnivores in the country using scientific and common names, and including more general search terms (mammal, vertebrate, or carnivore), for all available years up to and including 2015 and one early 2016 publication (S1 Appendix). We used Thomson Reuter’s Web of Science to identify indexed papers, and the Malaysian Citation Centre (http://www.myjurnal.my/public/browse.php) to search journals in all biological categories. For non-indexed Malaysian journals without online search capability we manually checked journal contents and excluded papers and records that were not from Peninsular Malaysia. Our final data set was derived from 85 published papers and reports (Fig 1, S2 Appendix) in the English language with carnivore records based on live captures, direct observations, signs, remote cameras, or road kills and other reported records from oldest to the most recent (1948 to 2014). Where publications did not provide coordinates of species records, we used an estimate of the center of the study area for georeferencing. We recorded the date of the study, location, and principal habitat types. Some studies were conducted in multiple geographic locations, thus the number of geographic locations (*n* = 89) exceeded the number of papers or reports (*n* = 85). Some geographic locations were surveyed more than once. We mapped recent (1991–2014) and older (prior to 1991) records by species, family, and IUCN Red List category. We used 1991 as a cut-off year because most major land-use changes have occurred since then. We used Kendall’s tau-b to explore associations among the number of records (all years) per species, body size, global (IUCN) and Peninsular Malaysia threat status [27, 28], and habitat breadth (number of different habitat types where a species was recorded). We weighted threat status for each species based on an interval scale of 1 (LC; least concern), 2 (NT; near threatened), 3 (VU; vulnerable), and 4 (EN or CE; endangered or critically endangered, respectively; see Table 1). We tested the hypothesis that threat status was negatively correlated with habitat breadth. We assessed eight broad habitat types reported in the literature (S2 Table) and used species with ≥8 records to assess associations with habitat breadth. Because riparian habitats were nested within most other habitats, they were not considered a separate habitat type for this analysis.

### Identifying priority conservation areas

We used the georeferenced species data for the period 1948–2014 to identify clusters of locations (i.e., hotspots) with carnivore assemblages for which conservation priorities were high [54,55]. Most studies identified in our review were suitable for this objective because they were broad-based mammal surveys. However, we excluded 25 papers where carnivore species records could not be linked with identifiable locations (a study area or geographic coordinate), or where records were duplicates from other publications. Thus, we used data from 60 papers for the hotspot analysis (Fig 1, S2 Appendix).

Our primary aim was to identify regions in the Malay peninsula that had high concentrations of species that were globally threatened. Thus, for the hotspot analysis, we weighted conservation priority for each species according to IUCN Red List status [27] based on an interval scale of 1 (LC), 2 (NT), 3 (VU), and 4 (EN or CE) as previously described. Using this scale value as a weighting factor, we calculated the Getis-Ord Gi* statistic in ArcGIS, which is a *z* score that provides a spatial statistic of where high or low values of the weighting factor occur [54]. This approach allowed us to identify areas where species of greater (high *z* scores; hotspots) or lower (low *z* scores; coldspots) global conservation concern were concentrated, which helped reduce potential bias due to where surveys were conducted [56]. To calculate the *z* scores, we used inverse-squared Euclidean distances to measure spatial relationships among the values of the weighting factor. This relationship allowed nearby carnivore observations to have greater influence on computations for a target location than observations further away, with the influence declining as a quadratic function of distance. The largest distance between two nearest species records was 85 km so we used that distance as a search radius to ensure that any unique survey location had at least one neighboring survey location. We used a kernel density estimator in ArcGIS, again with a search radius of 85 km, to create a continuous surface map of the *z* scores.

Finally, we examined relationships between the *z* scores and the landscape variables to gain insights into which landscape gradients may be associated with areas where carnivore species with high conservation rankings were concentrated as opposed to depleted. We examined whether the *z* scores were associated with the following environmental and anthropogenic landscape gradients: elevation, natural land cover, human population density, proximity to nearest town or village, and density of primary roads (S1 Dataset). We obtained elevation (m) data from the Consortium for Spatial Information (http://srtm.csi.cgiar.org/). We reclassified land-cover data from the Global Land Cover Database (http://forobs.jrc.ec.europa.eu/products/glc2000/legend.php) into a binary layer to represent all natural land cover types, excluding urban, cultivated, and managed areas. We then used a neighborhood analysis to calculate the proportion of natural land cover within a radius of 15 km. We chose 15 km to reflect the large scale of our analysis and to ensure that values covered the full range of very low up to 100% natural land cover. We obtained human population data (counts per 30-arc grid cell, or approximate density/km^2^) from a Global Population Distribution database (http://www.ciesin.org/). We calculated proximity to the geographic center of the nearest town or village digitized from Google Maps. Finally, using the line density function in ArcGIS, we calculated density of improved roads (km/km^2^; digitized from Google Maps) based on a moving window with a 15-km radius. Land cover and human population data were from 2000, which was the approximate mid-point of the period during which most carnivore observations were recorded. In addition to these environmental and anthropogenic variables, we considered a variable that may have affected the sampling distribution, namely proximity to the capital, Kuala Lumpur. Because of logistical considerations, many early surveys were conducted in relatively close proximity (~100 km) to the capital (we used the GPS coordinates of the headquarters of the Department of Wildlife and National Parks as our reference point). This area has relatively high densities of improved roads, therefore we added an interaction effect between road density and proximity to headquarters to every model to account for potential sampling bias. Given the large spatial scale of our assessment, we set the resolution of all data layers to 30-arc seconds for Peninsular Malaysia.

To explore potential relationships between the Getis-Ord Gi* *z* scores and landscape variables, we used ordinary least squares linear regression in ArcGIS to examine a set of models with different combinations of the environmental and anthropogenic variables to assess their relative influence. We used proximity to Kuala Lumpur, improved road density and their interaction as the basis for model building, to account for spatial sampling biases and reduce spatial autocorrelation [57]. We used the bias-corrected Akaike’s information criterion (AIC_c_) for model selection and considered models within 2 ∆AIC_c_ values to be parsimonious [58]. To reduce skewness in the data, we log-transformed human population and proximity to Kuala Lumpur and square-root transformed density of improved roads. We tested for normal distribution of residuals using the Jarque-Bera statistic. We used Koenker's studentized Breusch-Pagan statistic to determine if explanatory variables had a consistent relationship with Getis-Ord Gi* *z* scores in geographic space and data space. If this test was significant, we calculated robust standard errors, *t*-values, and probabilities for beta values. Finally, we tested whether model residuals showed spatial autocorrelation based on Moran’s I statistic.

## Results

### Records of distribution and habitat

**Fig 2:**
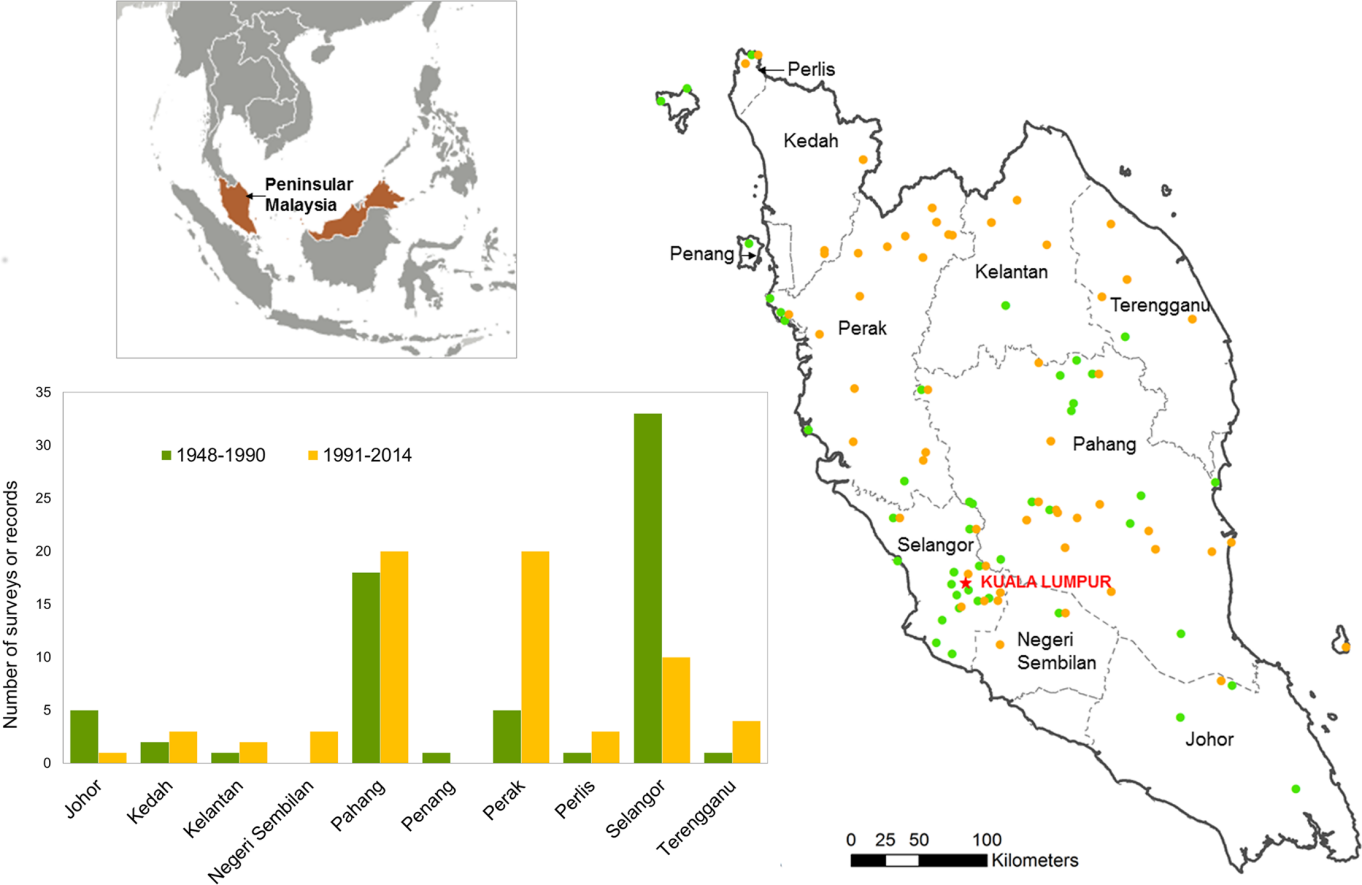
Distribution of surveys (*n* = 133) of carnivores among different states in Peninsular Malaysia with records collected during 1948–2014. Data were based on 60 published papers and reports that used conventional trapping, direct observation, signs, remote cameras, or road kills. Some publications compiled data from several surveys and some geographic locations were surveyed more than once. Boundary layer: U.S. State Department, Humanitarian Information Unit (modified from Global Large Scale International Boundary Polygons). Inset map: U.S. Central Intelligence Agency (The World Factbook).

**Fig 3:**
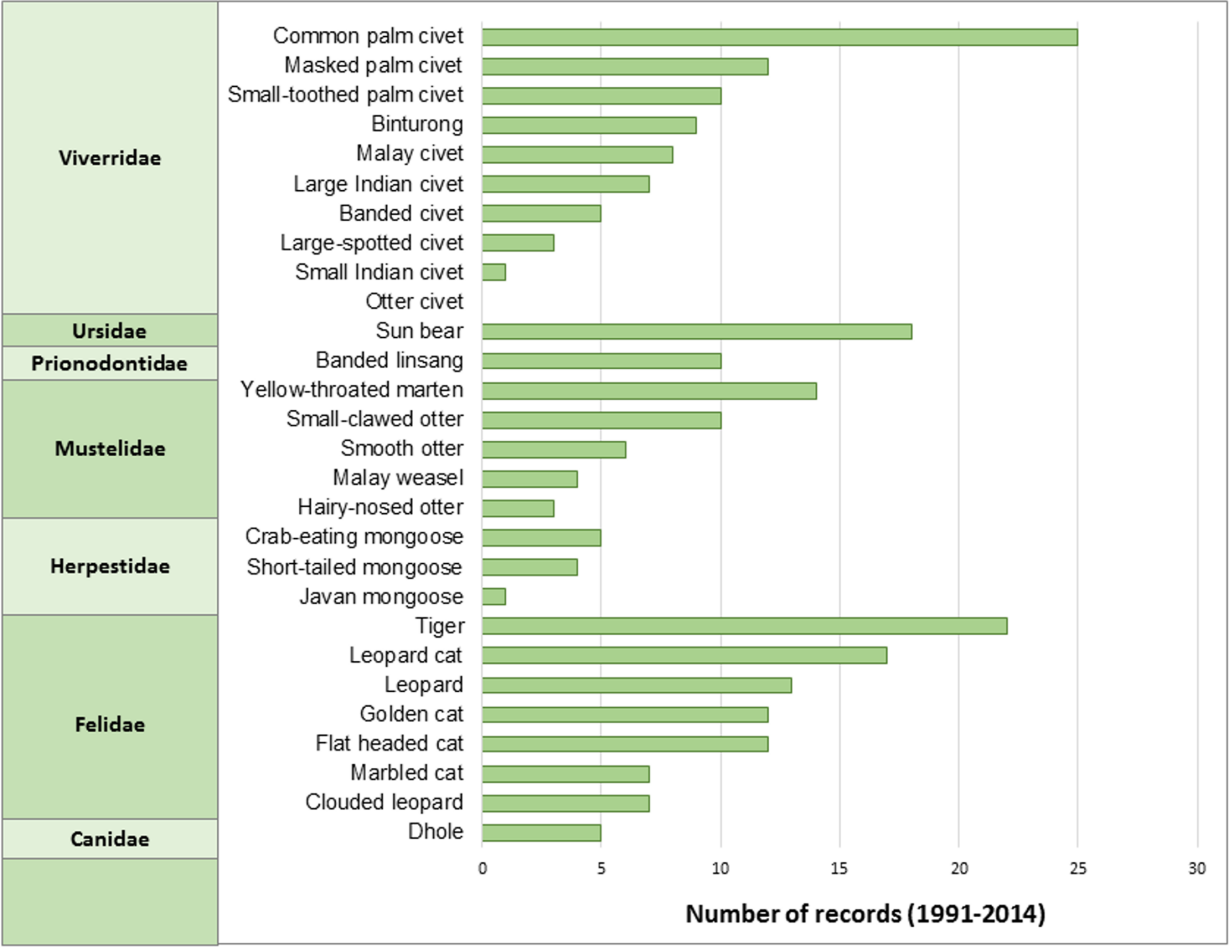
Number of records of Carnivora species in Peninsular Malaysia. Data were obtained from surveys that used conventional trapping, direct observation, sign, remote cameras, or road kills collected during 1991–2014. Species are grouped by family and ranked by number of records.

Observation records spanned the period 1948–2014 with 96% collected during the last 50 years and 50% collected after 1991 (Fig 2, S2 Table). We mapped all survey locations by family and species (S1–S4 Figs) and by threat category (S5–S7 Figs). Recent survey records (i.e., since 1991) in largely primary rainforest in northern Perak revealed high carnivore species richness. In Selangor, 75% of carnivore records preceded 1991, thus fewer surveys may have influenced the relative paucity of recent versus older carnivore records (S1–S7 Figs). Records were few (<5) for 10 species, almost all of which were small to medium-sized carnivores (Fig 3) and there were no recent records of the endangered otter civet (*Cyanogale bennetii*). The number of records tended to be greater with species’ body size (Kendall’s tau-b = 0.24, *z* = 1.78, *P* = 0.038), but not with IUCN global or Peninsular Malaysia conservation scores.

Surveys (or specimens collected) in forest reserves, wildlife reserves and national parks consisting mostly of dry-land forest comprised 75% of the reports. The remaining reports were from rice fields (12%), peat swamp/mangrove forest (6%), oil palm plantations (3%), mangrove forests (2%) and human inhabited areas (2%). We used carnivore species presence data from 89 geographic locations to examine habitat types associated with species records (S2 Table). Habitat breadth was associated with the number of records per species (Kendall’s tau-b = 0.554, *z* = 3.03, *P* = 0.001), but not with species’ IUCN global or Peninsular Malaysia conservation scores, nor with body size.

### Priority conservation areas

**Fig 4:**
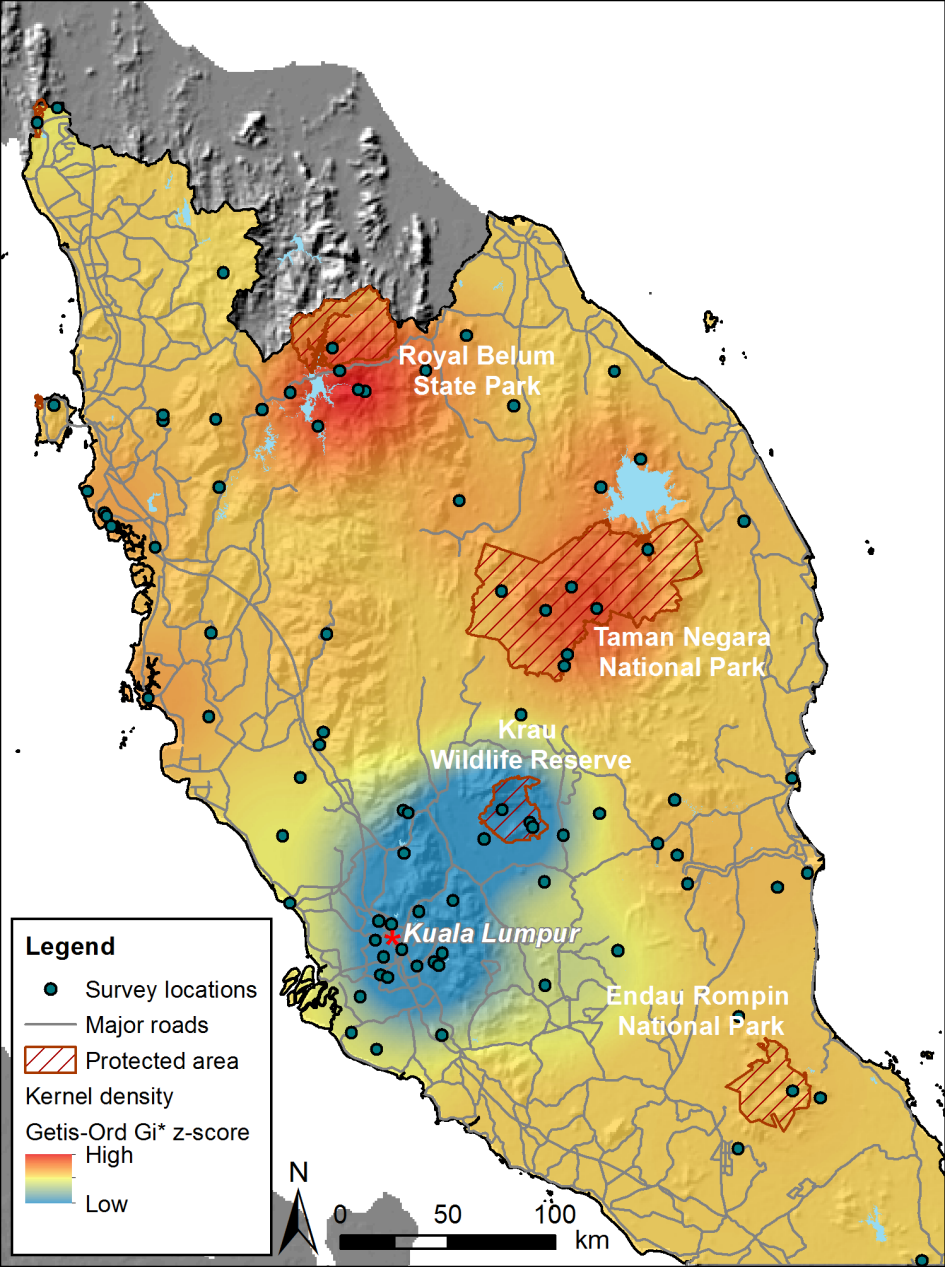
Locations of mammalian carnivore surveys and kernel density surface of Getis-Ord Gi* *z* scores of weighted ranking of IUCN red list categories for recorded species locations in Peninsular Malaysia, 1948–2014. Hillshade layer derived from Shuttle Radar Topography Mission (STRM) 90-m Digital Elevation Data from Consultative Group on International Agricultural Research (CGIAR) and reprinted under a CC BY license, with permission from International Center for Tropical Agriculture (CIAT), original copyright 2004. Protected areas mentioned in the text are labeled; reprinted from World Database on Protected Areas (http://www.protectedplanet.net) under a CC BY license, with permission from the United Nations Environmental Programme-World Conservation Monitoring Centre, original copyright 2010.

A region in the northeastern portion of the peninsula had the greatest concentration of carnivores with high conservation status, within which two areas were particularly prominent: the forest complex associated with Royal Belum State Park in the northern portion of this region and, southeast of it, an area associated with Taman Negara National Park (Fig 4). Notably, we also identified a concentrated area with carnivore observations and diversity associated with the southern half of Selangor and the adjacent region in Pahang, including Krau Wildife Reserve, but the presence of carnivores with high conservation status was much lower compared with other areas.

Model selection of ordinary least squares regressions showed the best-fitting model included elevation, proportion of natural land cover, improved road density, proximity to Kuala Lumpur, and the interaction between the latter 2 variables (adjusted *R*^2^ = 0.62; S1 Table). The second-best model was within 2 ∆AIC_c_ values and contained human population density as an additional variable. However, the 95% confidence interval of that variable overlapped zero so we focused our interpretation on the top model. The Jarque-Bera (*JB*) statistic indicated the residuals of the model did not deviate from normality (*JB* = 0.459, 2 df, *P* = 0.797). Getis-Ord Gi* *z* scores were negatively associated with elevation (β = -0.00124, SE = 0.00057, *t* = -2.169, *P* = 0.042) and positively associated with proportion of natural land cover (β = 1.899, SE = 0.711, *t* = 2.671, *P* = 0.009) and distance to Kuala Lumpur (β = 1.811, SE = 0.339, *t* = 5.344, *P* < 0.001). Thus, areas where observations of species with higher conservation ranks were spatially clustered generally coincided with areas at lower elevations, with greater proportion of natural land cover, and tended to be more distant from Kuala Lumpur. Human population density and proximity of the nearest town or village did not show an association with the Getis-Ord Gi* *z* scores. There was some evidence of spatial autocorrelation among the residuals (Moran’s I = 0.461, *z* = 2.018, *P* = 0.044).

## Discussion

Peninsular Malaysia contains possibly the greatest number of native species of Carnivora within Sundaland, and more than half are globally threatened or near threatened. Using data compiled from the first comprehensive review of publications with carnivore records, we identified two regions that overlapped with protected areas, Taman Negara National Park and Royal Belum State Park, as hotspots for carnivore species of greatest conservation concern.

Both these protected areas are considered priority regions for tiger conservation in Malaysia [59]. Established in 1938, Taman Negara (4343 km^2^) is Malaysia’s oldest national park [60] and comprises portions of the states of Pahang, Terengganu, and Kelantan. It contains Malaysia’s largest continuous tract of primary forest, of which nearly 60% consists of low elevation (75–300 m) rainforest. Royal Belum State Park, however, was gazetted in 2007 [61] and is part of the Belum-Temengor Forest Complex (3546 km^2^) located in northern Perak; it shares its northern boundary with Thailand, where it connects with two protected areas, Hala Bala Wildlife Sanctuary and Bang Lang National Park. The combined extent of protected areas and forest reserves in this forest complex, which consists of lowland and hill dipterocarp forests from 130 to 1500 m, may rival that of Taman Negara [62]. The number of carnivore species reported in Taman Negara and Belum-Temengor were 19 and 22, respectively, each with eight threatened and five near-threatened species.

A crucial finding was the relative scarcity of reports of carnivores of conservation concern in the southwestern region of the Peninsular encompassing the state of Selangor and the adjacent region in the state of Pahang, despite frequent surveys in that area. The surveys were conducted within a 50- to 60-km radius of Kuala Lumpur, where several small forest reserves and areas (12–200 ha) of secondary forest have existed within the city limits for decades, with more extensive lowland and hill dipterocarp forests in peri-urban areas [63]. Surveys in this region occurred over a long time span, with over half the records collected prior to 1991. The distinct paucity of records of carnivores of conservation concern suggests that many of these species cannot persist in small fragmented habitats, or even in larger extents of habitats close to urbanization. Krau Wildlife Reserve (603 km^2^), situated within a 1556-km^2^ forested area [64], was the largest protected area in this coldspot. The forest is surrounded by agriculture and settlements, but its northeastern boundary is <50 km south of the large forested landscape of Taman Negara and consequently considered a secondary priority site for tiger conservation in Malaysia [65]. Carnivore species richness in Krau (*n* = 20) resembled Belum-Temengor and Taman Negara, although with fewer threatened (*n* = 5) and near-threatened species (*n* = 4).

Carnivore hotspots were associated with large extents of natural land cover, lower elevations, and greater distances from Kuala Lumpur, which is situated within the state of Selangor. Selangor (800,000 ha), the most populous state in Malaysia with 5.8 million people [66], has the highest per capita GDP and has experienced the most rapid growth in the manufacturing sector in the last five decades. Urban and agricultural development has been responsible for most of the state’s change in land use with the expansion of oil palm plantations at the expense of peat swamp forest [42, 67]. Considering that 75% of the surveys in Selangor were conducted before 1991 and our human population and land use data were derived more recently, the status of carnivore populations in this state may be more critical than the data suggest.

A common consequence of urbanization and development is habitat fragmentation and the extirpation of large apex predators. Laidlaw’s [68] survey of seven sites (70 to >10,000 ha) in Peninsular Malaysia suggested that large tracts of natural forest were the most important predictor of mammal species richness and large carnivore presence. Woodroffe [16] demonstrated a strong positive relationship between reserve size and the persistence of large carnivores and concluded that smaller habitat patches increased the potential for human-carnivore conflicts with subsequent extirpation of local carnivore populations. Many small and mid-sized carnivores also rely on larger habitat patches suggesting that factors other than body size, such as resource specialization, behavior, and social structure, play an important role in this dependency [16, 69]. Smaller habitat patches could mean the loss of suitable habitat, new barriers to movement, or competition with species better adapted to disturbed environments [70]. Proximity to urbanization and primary roads, even where habitat is sufficiently large, limits dispersal and enhances the risk of road mortality and illegal hunting [71–73].

Low-elevation habitats with natural forest cover may be one of the most valuable habitats for carnivores in tropical regions. We found that all but two species of Carnivora were reported in lowland forests (S2 Table). Notably, the number of species of Carnivora reported in lowland swamp forests (*n* = 17) was high, considering the relatively few surveys (*n* = 16). In Southeast Asia, lowland equatorial forests support the vast majority of species [46] and in Peninsular Malaysia, lowland forests support almost 90% of mammal species with 61% occurring only in lowland and hill forests below 1000 m [74]. Malaysia has lost nearly 40% of its original forest cover [75] and recent annual deforestation rates in the peninsula (0.9% annually from 2000 to 2010 [46]) show little sign of abatement.

With the exception of the otter civet (one record in 1987), records since 1991 exist for the remaining 27 species in the peninsula. Records were few for nine species, mostly small carnivores, including four species of Viverridae and the three species of Herpestidae native to the Malay Peninsula. We found only one record of the Javan mongoose (*Herpestes javanicus*) and one of the small Indian civet (*Viverricula indica*) since 1991; these species are neither globally threatened nor near threatened. Conversely, records were greater for larger species such as the tiger, sun bear (*Helarctos malayanus*), and leopard (*Panthera pardus*). In an extensive review, Brooke et al. [76] reported a strong association between body size and research effort in the Carnivora, with the Viverridae and Herpestidae among the four least-studied of the carnivore families. Larger species leave more definitive signs and range over larger areas, increasing the probability of detection. Also, the rarely recorded Javan mongoose and small Indian civet favor open, less forested habitats [77,78]; apart from rice fields, these habitats are rare in Peninsular Malaysia. The dearth of ecological studies on smaller carnivores in peninsular Malaysia may predispose them to early extinction, when efforts for their conservation are less costly than for large-bodied species, and more likely to succeed [79].

Large body size confers greater vagility and the ability to use a wide array of habitats, but we found no association between habitat breadth and body size. Also, species that use a wide range of habitats may be more tolerant of habitat loss and fragmentation [80]. Although there may be some sampling bias given that species with more records were reported in more habitats, habitat breadth was not associated with global (IUCN) or local (Peninsular Malaysia) threat status. To illustrate, three small carnivores, the common palm civet (*Paradoxurus hermaphroditus*; least concern), the leopard cat (*Prionailurus bengalensis*; least concern), and the flat-headed cat (*Prionailurus planiceps*; endangered) were reported in a wide variety of habitats (S2 Table), including small forest patches in urban landscapes. The flat-headed cat is adapted for feeding on aquatic prey, thus the presence of wetland habitat, which is abundant in Peninsular Malaysia, may be more important for its persistence than forest cover. Locally, the flat-headed cat is considered near threatened [28], in contrast with its global endangered status [27], which may reflect its ability to persist in a variety of habitats associated with freshwater.

We acknowledge several caveats in our study. Despite our attempt to obtain as complete a set of published studies for our analysis as possible, at least three papers with nine additional records of leopard [81, 82] and one record of a flat-headed cat [83] escaped our attention. Of these 10 records, including 29 recently released records of threatened and near-threatened carnivores [84–87], 82% occurred within the hotspots identified in our analysis, confirming the importance of these regions for carnivore conservation. We caution, however, that despite demonstrating distinct landscape associations with the distribution of carnivores as weighted by their conservation rankings, we could not fully account for spatial autocorrelation and our data were not derived from standardized, probabilistic, or systematic coverage of the entire peninsula. Thus, our inference is weaker in areas with fewer surveys and published records. For example, the data included few surveys for the southern region of the peninsula, including the Endau Rompin Forest Complex (~2389 km^2^). This area comprises substantial low-elevation rainforest with the potential to support a diversity of indigenous carnivores despite its highly fragmented surroundings and poor connectivity with large, forested landscapes [65]. A recent remote camera survey reported the presence of six felid species, including tigers [88].

## Conclusion

Peninsular Malaysia supports several species of globally threatened carnivores and our study underscores the importance of natural forest cover for their persistence. We show that carnivores of greatest conservation concern are less likely to persist in small, fragmented habitats or habitats close to urban areas. Recent (2000–2012) changes in global forest cover indicate that Malaysia lost 14% of its forest cover, a rate of loss that exceeded any other country [89]. Oil palm and industrial timber plantations replaced most of the lost forest [90] and trends point to their continued expansion. Surveys and targeted ecological studies of carnivores in habitat types other than primary and secondary forests will thus be important to elucidate their status and capacity to persist in the face of progressive habitat alteration. Recent studies in oil palm estates and commercial forest plantations suggest that these altered habitats may serve as ecological corridors and shelter valuable elements of biodiversity [91–93], but primarily when interspersed with large (>1000 ha) stands of natural, secondary forest [94]. Ultimately, addressing the two key threats of poaching and habitat loss will be crucial for the persistence of Malaysia’s most threatened carnivores and consequently the broader ecological communities that carnivores influence.

## Supporting information

S1 Appendix Search terms and sources for carnivore records and habitats in Peninsular Malaysia. (PDF)

S2 Appendix Records of Carnivora by species, locations, and year. (XLSX)

S1 Dataset Geo-referenced TIFF files for spatial data layers used in landscape analysis. (ZIP)

S1 Fig Recent (1991–2014) and older (1948–1990) records within the family Felidae in Peninsular Malaysia. Boundary layer: U.S. State Department, Humanitarian Information Unit (modified from Global Large Scale International Boundary Polygons). Inset map: U.S. Central Intelligence Agency (The World Factbook).
(DOCX)

S2 Fig Recent (1991–2014) and older (1948–1990) records of species within the families Prionodontidae (banded linsang), Ursidae (sun bear), and Canidae (dhole) in Peninsular Malaysia. Boundary layer: U.S. State Department, Humanitarian Information Unit (modified from Global Large Scale International Boundary Polygons). Inset map: U.S. Central Intelligence Agency (The World Factbook).
(DOCX)

S3 Fig Recent (1991–2014) and older (1948–1990) records of species within the families Mustelidae and Herpestidae in Peninsular Malaysia. Boundary layer: U.S. State Department, Humanitarian Information Unit (modified from Global Large Scale International Boundary Polygons). Inset map: U.S. Central Intelligence Agency (The World Factbook).
(DOCX)

S4 Fig Recent (1991–2014) and older (1948–1990) records of species within the family Viverridae in Peninsular Malaysia. Boundary layer: U.S. State Department, Humanitarian Information Unit (modified from Global Large Scale International Boundary Polygons). Inset map: U.S. Central Intelligence Agency (The World Factbook).
(DOCX)

S5 Fig Distribution of endangered species in Peninsular Malaysia. Solid icons represent recent (1991–2014) records and unfilled icons represent older (1948–1990) records. Boundary layer: U.S. State Department, Humanitarian Information Unit (modified from Global Large Scale International Boundary Polygons). Inset map: U.S. Central Intelligence Agency (The World Factbook). IUCN changed the status of the Malaysian tiger from endangered to critically endangered in 2015.
(DOCX)

S6 Fig Distribution of vulnerable species in Peninsular Malaysia. Solid icons represent recent (1991–2014) records and unfilled icons represent older (1948–1990) records. Boundary layer: U.S. State Department, Humanitarian Information Unit (modified from Global Large Scale International Boundary Polygons). Inset map: U.S. Central Intelligence Agency (The World Factbook).
(DOCX)

S7 Fig Distribution of near-threatened species in Peninsular Malaysia. Solid icons represent recent (1991–2014) records and unfilled icons represent older (1948–1990) records. Boundary layer: U.S. State Department, Humanitarian Information Unit (modified from Global Large Scale International Boundary Polygons). Inset map: U.S. Central Intelligence Agency (The World Factbook).
(DOCX)

S1 Table Model selection results to identify landscape variables associated with spatial clustering of carnivore records based on weighted ranking of IUCN red list categories. (PDF)

S2 Table Carnivora species reported in Peninsular Malaysia and associated habitats, 1948–2014. (PDF)
