# Supplementary material for: Carnivore hotspots in Peninsular Malaysia and their landscape attributes
Source: PLoS One. 2018 Apr 4;13(4):e0194217. doi: 10.1371/journal.pone.0194217 (PMC5884492; doi:10.1371/journal.pone.0194217)
Supplement: S1 Appendix — (PDF) [file pone.0194217.s001.pdf]

S1 Appendix. Search terms and sources for carnivore records and habitats in Peninsular Malaysia.

Search Terms

Malaysia, carnivore, mammal, yellow-throated marten, weasel, Mustela, Viverricula, Viverra, sun bear, Prionailurus, Panthera, Catopuma, Neofelis, Pardofelis, marbled cat, leopard, felid, otter, mongoose, dhole, civet, binturong, banded linsang, Prionodon.

References

1. Abdul JB. A short note on the otter civet (*Cyanogale bennetii*). Journal of Wildlife and Parks. 1987;6: 87.
2. Azhar B, Lindenmayer DB, Wood J, Fischer J, Zakaria M. Ecological impacts of oil palm agriculture on forest mammals in plantation estates and smallholdings. Biodiversity Conservation. 2014;23: 1175–1191.
3. Azlan JM, Sharma DSK. Camera trapping the indochinese tiger, *Panthera tigris corbetti*, in a secondary forest in Peninsular Malaysia. Raffles Bulletin of Zoology 2003;51: 421–427.
4. Azlan JM, Sharma DSK. Mammal diversity and conservation in a secondary forest in Peninsular Malaysia. Biodiversity and Conservation. 2006;15: 1013–1025.
5. Azlan JM, Sharma DSK. The diversity and activity patterns of wild felids in a secondary forest in Peninsular Malaysia. Oryx. 2006;40 :36–41.
6. Baker N. New Records of Hairy-Nosed Otter (*Lutra sumatrana*) in Peninsular Malaysia. IUCN Otter Specialist Group Bulletin. 2013;30: 112–118.
7. Burhanuddin HJMN. Preliminary study on food preference of Lutra perspicillata and Aonyx cinerea in Tanjung Piandang, Perak. Journal of Wildlife and Parks. 1989;8: 47–51.
8. Burhanuddin HJMN, Jani Z, Hasan K. A preliminary analysis on large mammals distribution patterns and habitats in Johore from 1992–1998. Journal of Wildlife and Parks. 1997;16: 110–119.
9. Burhanuddin HJMN, Norizan A. A survey on the distribution of otters in Pulau Pinang and Perlis. Journal of Wildlife and Parks. 1990.9:53–58.
10. Burhanuddin HJMN, Norizan A, Sukigara S. Estimation On The Density Of Common Palm Civet (*Paradoxurus hermaphroditus*) In Oil Palm Plantation At Kuala Gula, Perak. Journal of Wildlife and Parks. 1994;13: 1–7.
11. Ean TP. 2010. A checklist of mammals in Tasek Bera Ramsar Site, Pahang. Journal of Wildlife and Parks. 26:71–77.
12. Ean TP, Norazlinda A, Rosedan M, Yazid AZ, Magintan D. New locality records of the monkey-footed rat (*Pithecheir parvus*) and crab-eating mongoose (*Herpested urva*) in Kuala Lompat, Krau Wildlife Reserve, Pahang, Malaysia through cage-trapping surveys. Journal of Wildlife and Parks. 2014;28: 139–140.
13. Foster-Turley P. Conservation Aspects of the Ecology of Asian Small-Clawed and Smooth Otters on the Malay Peninsulas IUCN Otter Spec. Group Bull. 1992;7: 26–29. Available from: http://www.ecologyandsociety.org/vol14/iss1/art21/
14. Gumal M, Salleh A, Yasak M, Horng LS, Lee BPYH, Pheng LC. et. al. Small-medium wild cats of Endau Rompin Landscape in Johor, Peninsular Malaysia. CATnews Special Issue. 2014;8: 10–18.
15. Hamirul M, Wong CCT, Mohamed A, Lau CF, S. W. Mohamed SW, E. S. Siwan ES, Rayan DM. Recent records of large-spotted civet *Viverra megaspila* from Peninsular Malaysia. Small Carnivore Conservation. 2015;52 & 53: 74–83.
16. Hedges L, Clements GR, Aziz S, Yap W, Laurance S, Goosem M, Laurance W. 2013. Small carnivore records from a threatened habitat linkage in Terengganu, Peninsular Malaysia. Small Carnivore Conservation. 49:9–14.

17. Hedges L, Lam WY, Campos-Arceiz A, Rayan DM, Laurance WF, Latham CJ, Saaban S, Clements GR. Melanistic leopards reveal their spots: Infrared camera traps provide a population density estimate of leopards in Malaysia. Journal of Wildlife Management. 2015;79: 846–853.
18. Jayaraj VK, Daud SHM, Azhar MI, Sah SAM, Mokhtar SI, Abdullah MT. 2013. Diversity and Conservation status of Mammals in Wang Kelian State Park, Perlis, Malaysia. Check List. 2013;9: 1439–1448.
19. Jennings AP, Veron G. Predicted distributions and ecological niches of 8 civet and mongoose species in Southeast Asia. Journal of Mammalogy. 2011;92 :316–327.
20. Jennings AP, Veron G. Predicted distributions and conservation status of two threatened SouthEast Asian small carnivores: the banded civet and Hose's civet. Mammalia. 2013;77: 261–271.
21. Jennings AP, Veron G. Predicted distributions, niche comparisons, and conservation status of the spotted linsang (*Prionodon pardicolor*) and banded linsang (*Prionodon linsang*). Mammal Research. 2014;60: 107–116.
22. Jennings AP, Zubaid A, Veron G. Home ranges, movements and activity of the short-tailed mongoose (*Herpestes brachyurus*) on Peninsular Malaysia. Mammalia. 2010a;74: 43–50.
23. Jennings AP, Zubaid A, Veron G. Ranging behaviour, activity, habitat use, and morphology of the Malay civet (*Viverra tangalunga*) on Peninsular Malaysia and comparison with studies on Borneo and Sulawesi. Mamm. Biol. 2010b;75: 437–446.
24. Kassim H, Aziz C, Topani R, Muhamad M, Wahid S, Yusof E. Large Mammal Survey at Krau Wildlilfe Reserve, Pahang. Journal of Wildlife and Parks. 1999;17: 89–95.
25. Kawanishi K, Sahak AM, Sunquist M. Preliminary analysis on abundance of large mammals at Sungai Relau, Taman Negara. Journal of Wildlife and Parks. 1999;17: 62–82.
26. Kawanishi K, Sunquist M. Possible new records of fishing cat from Peninsular Malaysia. Cat News. 2003;39: 3–5.
27. Kawanishi K, Sunquist M. Conservation status of tigers in a primary rainforest of Peninsular Malaysia. Biological Conservation. 2004;120: 329–344.
28. Laidlaw RK. Effects of habitat disturbance and protected areas on mammals of peninsular Malaysia. Conservation Biology. 2000;14: 1639–1648.
29. Lim BL. The banded linsang and the banded musang of West Malaysia. Malayan Nature Journal. 1973;26: 105–111.
30. Lim BL. The distribution, food habits and parasite patterns of the leopard cat (*Prionailurus bengalensis*) in Peninsular Malaysia. Journal of Wildlife and Parks. 1999;17: 17–27.
31. Lim BL. The distribution and food habits of *Pardofelis marmorata* in Peninsular Malaysia. Journal of Wildlife and Parks. 2001;19: 125–127.
32. Lim BL. Distribution and food-habits of the Asiatic golden cat (*Catopuma temminckii*) in Peninsular Malaysia. Journal of Wildlife and Parks. 2002;20: 43–48.
33. Lim BL. Critical habitats for the survival of Malayan mammals in Peninsular Malaysia. Journal of Science and Technology in the Tropics. 2008;4: 27–37.
34. Lim, BL, Rahman OA. Observations on the habits in captivity of two species of wild cats, the leopard cat and the flat-headed cat. Malays Nat Hist J. 1961;15: 8–51.
35. Lim BL, Anan S. Small mammals of Taman Negara. In Y. H. Sen (Eds.) A special issue to commemorate the golden jubilee of Taman Negara. Journal of Wildlife and Parks, Malaysia. 1990;X: 148–152.
36. Lim BL, Hussein N, Ratnam L. A Study of the Vertebrate Fauna in the Tasek Chini Nature Reserve, Pahang in Relation Use. Malayan Nature Journal. 1999a;53: 217–238.
37. Lim BL, Khan M, Chai K, Lim C. Pre-logging survey of herpetological and mammal fauna at Lakum Forest Reserve, Raub, Pahang, Malaysia. Science & Technology 2008;4: 99–116.
38. Lim BL, Lim Kkp, Yong HS. The terrestrial mammals of Pulau Tioman, peninsular Malaysia, with a catalogue of specimens at the Raffles Museum, National University of Singapore. Raffles Bulletin of Zoology 1999b;47: 101–123.
39. Lim BL, Majid R, Norsham Y. Studies on the mammal fauna of Bukit Kutu Wildlife reserve, Hulu Selangor. Journal of Wildlife and Parks 1999c;17: 1–16.

40. Lim BL, Nazim T. The flat-headed cat, *Prionailurus planiceps* (Vigors & Horsefield, 1827) of Peninsular Malaysia: distribution, food habits, and parasite pattern. Journal of Wildlife and Parks 2005;22: 1–9.
41. Lim BL, Ratnam L, Anan S. Study of the small mammals in Taman Negara with special reference to the rat lung-worm. Journal of Wildlife and Parks. 1989;8: 17–30.
42. Lim BL, Ratnam L, Francis C. 1995. Vertebrate fauna. Herpetofauna, birds and mammals. Pages 34–54 *in* S. Lee, editor. A guide book to Pasoh. FRIM technical information handbook.
43. Lim BL, Yong D, Tharmalingam M, Shin C, Lim C. Vertebrate species diversity in the Broga and Sungai Lallang Forest Reserves, Hulu Langat, Selangor, peninsular Malaysia. Journal of Science and Technology in the Tropics. 2009;5: 87–99.
44. Lynam AJ, Laidlaw R, Wan Noordin WS, Elagupillay S, Bennett EL. Assessing the conservation status of the tiger *Panthera tigris* at priority sites in Peninsular Malaysia. Oryx. 2007;41: 454–462.
45. Magintan D, Ahmad MA, Ismail A, Rasdi I. Observation of dhole (Cuon alpinus) at Sungkai Wildlife Reserve, Perak, Malaysia. Journal of Wildlife and Parks. 2014;29: 69–72.
46. Magintan D, Rufino M, Cosmas N, Dennis T. Some evidences of Sumatran rhinoceros presence in Temengor Forest Reserve, Perak. Journal of Wildlife and Parks. 2010;26: 5–10.
47. Medway, L. 1969. The wild mammals of Malaya and offshore islands including Singapore.
48. Medway, L. 1972. The Gunung Benom expedition, 1967. 6. The distribution and altitudinal zonations of birds and mammals on Gunung Benom. Bulletin of the British Museum (Natural History) Zoology 23:106–154.
49. Mohamad SW, Rayan DM, Christopher WCT, Hamirul M, Mohamed A, Lau CF, Siwan ES. The first description of population density and habitat use of the mainland clouded leopard Neofelis nebulosa within a logged-primary forest in South East Asia. Population Ecology. 2015;57: 495–503.
50. Muul I, Lim BL. Ecological and Morphological Observations of *Felis planiceps*. Journal of Mammalogy. 1970;51: 806–808.
51. Muul I, Lim BL. New Locality Records for Some Mammals of West Malaysia. Journal of Mammalogy. 1971;52: 430–437.
52. Nazeri M, Jusoff K, Madani N, Mahmud AR, Bahman AR, Kumar L. Predictive modeling and mapping of Malayan Sun Bear (*Helarctos malayanus*) distribution using maximum entropy. PLoS One 2012; 7: 1-9. Available from: https://doi.org/10.1371/journal.pone.0048104.
53. Nazeri M, Kumar L, Jusoffa K, Bahaman AR. Modeling the potential distribution of sun bear in Krau wildlife reserve, Malaysia. Ecological Informatics 2014;20: 27–32.
54. Norsham Y, Bernard H, Chew K, Yap M, Yong H, Lim BL. A survey of mammals in the northern part of Belum Forest Reserve, Perak, Peninsular Malaysia. Malayan Nature Journal. 2000;54: 233–244.
55. Norsham Y, Ong T. 2001. Vertebrate fauna of compartment 14, Sungai Lalang Forest Reserve, Selangor. Journal of Wildlife and Parks. 2001;19: 99–108.
56. Norsham Y, Shariff F, Norhayati A, Nordin M, Lim BL. Pre-logging survey of mammal fauna at Sungai Weng sub-catchment, Ulu Muda Forest Reserve, Kedah. Journal of Wildlife and Parks. 1999;17: 28–43.
57. Ratnam L, Lim BL, Hussein NA. 1995. Mammals of the Sungai Singgor area in Temengor Forest Reserve, Hulu Perak [Perak], Malaysia. Malayan Nature Journal (Malaysia). 1995;48: 409–423.
58. Ratnam L, Saharudin A, Shariff SM. Ecological studies on the small mammals of the Air Hitam Forest Reserve, Puchong, Selangor. Journal of Wildlife and Parks. 1987;87: 88–97.
59. Rayan DM. Tiger Monitoring Study in Gunung Basor Forest Reserve, Jeli, Kelantan. Unpublished Report. WWF-Malaysia, Petaling Jaya, Malaysia. 2007.
60. Rayan DM, Mohamed SW. New locality records of the crab-eating mongoose Herpestes urva in Peninsular Malaysia as revealed by camera-trapping. Small Carnivore Conservation. 2008;39: 25–28.
61. Rayan DM, Mohamed SW. The importance of selectively logged forests for tiger Panthera tigris conservation: a population density estimate in Peninsular Malaysia. Oryx. 2009;43: 48–51.
62. Rayan DM, Linkie M. Conserving tigers in Malaysia: a science-driven approach for eliciting conservation policy change. Biological Conservation. 2015;184: 18–26.

63. Reza MIH, Abdullah SA, Nor SB, Shukor Bin Md, Ismail, MH. Integrating GIS and expert judgment in a multi-criteria analysis to map and develop a habitat suitability index: A case study of large mammals on the Malayan Peninsula. Ecological indicators. 2013;34: 149–158.
64. Saharudin A. Notes on the Crab-eating mongoose from Peninsular Malaysia. Journal of Wildlife and Parks. 1990;9: 45–46.
65. Sasidhran S, Adila N, Hamdan MS, Samantha LD, Aziz N, Kamarudin N, Puan CL, Turner E, Azhar B. Habitat occupancy patterns and activity rate of native mammals in tropical fragmented peat swamp reserves in Peninsular Malaysia. Forest Ecology and Management 2016;363: 140–148.
66. Wilting A, Cord A, Hearn AJ, Hesse D, Mohamed A, Traeholdt C, et al. Modelling the Species Distribution of Flat-Headed Cats (Prionailurus planiceps), an Endangered South-East Asian Small Felid. PLos One. 2010;5: e9612 DOI: 10.1371/journal.pone.0009612.
67. Sebastian AJ. The hairy-nosed otter in Peninsular Malaysia. IUCN Otter Spec Group Bull. 1995;11: 3.
68. Sen YH. A special issue to commemorate the golden jubilee of Taman Negara. Journal of Wildlife and Parks, Malaysia. 1990;X: 1–152.
69. Shariff S. Some observations on otters at Kuala Gula, Perak and National Park, Pahang. Journal of Wildlife and Parks. 1984;3: 75–88.
70. Shariff S. The occurrence of otters in the rice fields and coastal islands; and the comparison of these habitats. Journal of Wildlife and Parks. 1985;4: 20–24.
71. Shariff S, Hamid R. Ectoparasitic acari of small mammals from montane area of Cameron Highlands, Pahang. Journal of Wildlife and Parks 1993;12: 49–60.
72. Sharma D. 1992. A survey of the fauna, flora and geological features at the Mata Ayer Forest Reserve and the proposed Bukit Wang Mu Forest Reserve, Perlis. Petaling Jaya: WWF Malaysia. Available from: http://repository.wwf.org.my/technical_reports/B/BiodiversityInPeninsularMalaysiaTransfrontierForestsACaseStudyAtMataAyerForestReserveFRAndProposedBukitWangMuFRPerlis.pdf. Cited 16 October 2017.
73. Sivasothi N, Burhanuddin HMN. 1994. A review of otters (Carnivora: Mustelidae: Lutrinae) in Malaysia and Singapore. Hydrobiologia. 1994;285: 151–170.
74. Syakirah S, Zubaid A, Lopez A, Prentice C, Azmin M, Mohd-Yusof A. A small-mammal survey at Tasek Bera, Pahang, Malaysia's first Ramsar site. Malayan Nature Journal. 2000;54: 31–41.
75. Topani R. Status and distribution of tiger in Peninsular Malaysia. Journal of Wildlife and Parks. 1990;9: 71–102.
76. Van Bree P, Khan M, Khan M. On a fishing cat, *Felis* (*Prionailurus*) *viverrina* Bennett, 1833, from continental Malaysia. Zeitschrift für Säugetierkunde. 1992;57: 179–180.
77. Veron G, Willsch M, Dacosta V, Patou ML, Seymour A, Bonillo C, et al. The distribution of the Malay civet *Viverra tangalunga* (Carnivora: Viverridae) across Southeast Asia: natural or human-mediated dispersal? Zoological Journal of the Linnean Society. 2014;170: 917–932.
78. Veron, G, Gaubert P, Franklin N, Jennings AP, Grassman LI. A reassessment of the distribution and taxonomy of the Endangered otter civet *Cynogale bennettii* (Carnivora : Viverridae) of South-east Asia. Oryx. 2006;40: 42–49.
79. Wells D. Notes on the distribution and taxonomy of Peninsular Malaysian mongooses (*Herpestes*). Natural History Bulletin of the Siam Society. 1989;37: 87–97.
80. Wells D, Francis C. 1988. Crab-eating Mongoose *Herpestes urva*, a mammal new to peninsular Malaysia. Malayan Nature Journal. 1988;42: 37–41.
81. Yatim SH. A preliminary survey on inventory habitat and wildlife in the Ulu Langat Forest Reserve and Sungai Dusun Game Reserve. Journal of Wildlife and Parks. 1983;2: 119–140.
82. Yatim SH. Survey of Mammals and Bird Species in Tanjung Hantu Forest Reserve, Perak, Gunung Machinchang Forest Reserve Pulau Langkawi, and Kuala Gula Bird Sanctuary, Perak. Journal of Wildlife and Parks. 1984;3: 18–36.
83. Yatim SH, Zainuddin B, Marsuki MI. Survey of mammal and bird species at eight game/forest reserves. Journal of Wildlife and Parks. 1986;5: 24–52.

84. Yatim SH, Zainudin B, Saaidun KA, Marzuki MI. Survey of mammal and bird species in six different habitats: mangrove, lowland dipterocarp, hill dipterocarp, upper dipterocarp, montane oak and montane ericaceous forest. Journal of Wildlife and Parks 1985;4: 67–91.
85. Yusof E. Sorenson KW. Krau Wildlife Reserve: protected area management experiences. Journal of Wildlife and Parks. 2000;18: 3–13.
