# Supplementary figures and images for: Carnivore hotspots in Peninsular Malaysia and their landscape attributes
Source: PLoS One. 2018 Apr 4;13(4):e0194217. doi: 10.1371/journal.pone.0194217 (PMC5884492; doi:10.1371/journal.pone.0194217)

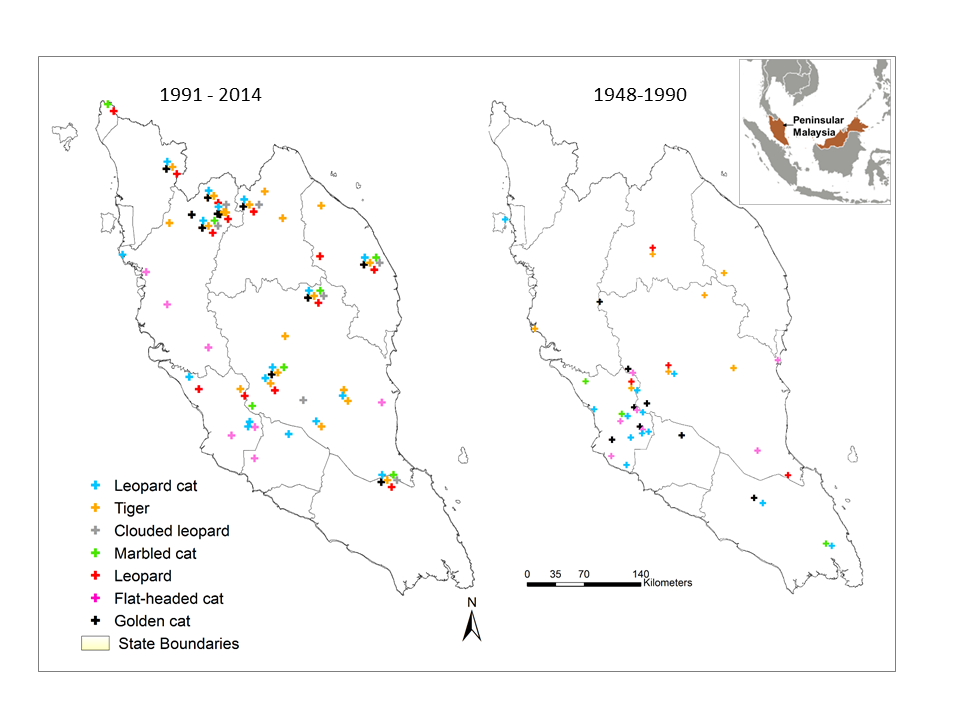


**S1 Fig.**

Supplement: S1 Fig — Boundary layer: U.S. State Department, Humanitarian Information Unit (modified from Global Large Scale International Boundary Polygons). Inset map: U.S. Central Intelligence Agency (The World Factbook). (DOCX) [file pone.0194217.s004.docx]

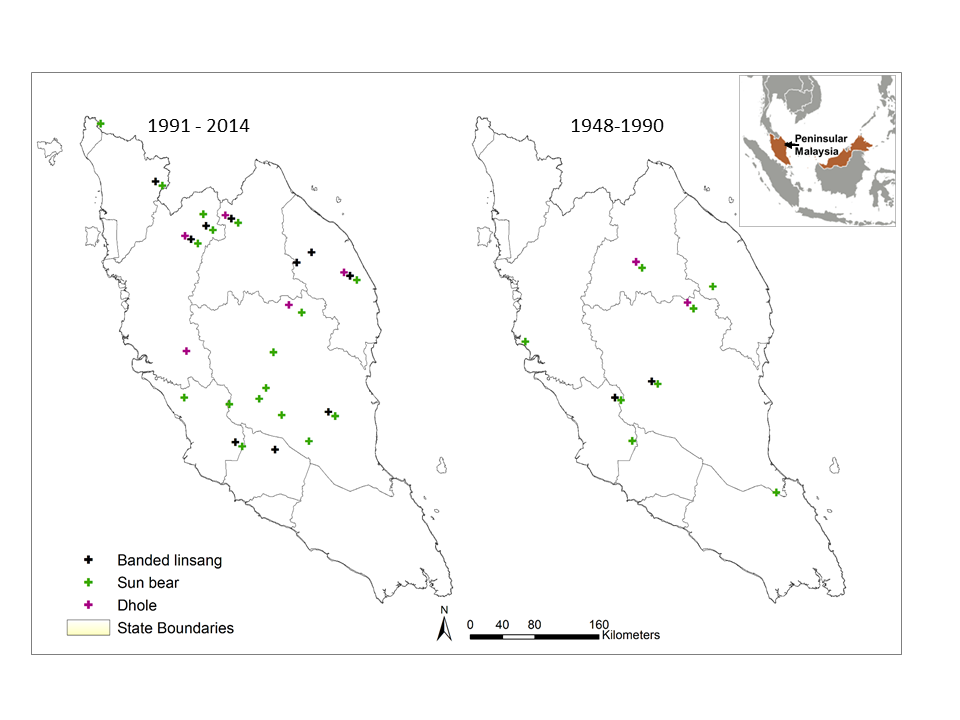


**S2 Fig.**

Supplement: S2 Fig — Boundary layer: U.S. State Department, Humanitarian Information Unit (modified from Global Large Scale International Boundary Polygons). Inset map: U.S. Central Intelligence Agency (The World Factbook). (DOCX) [file pone.0194217.s005.docx]

**
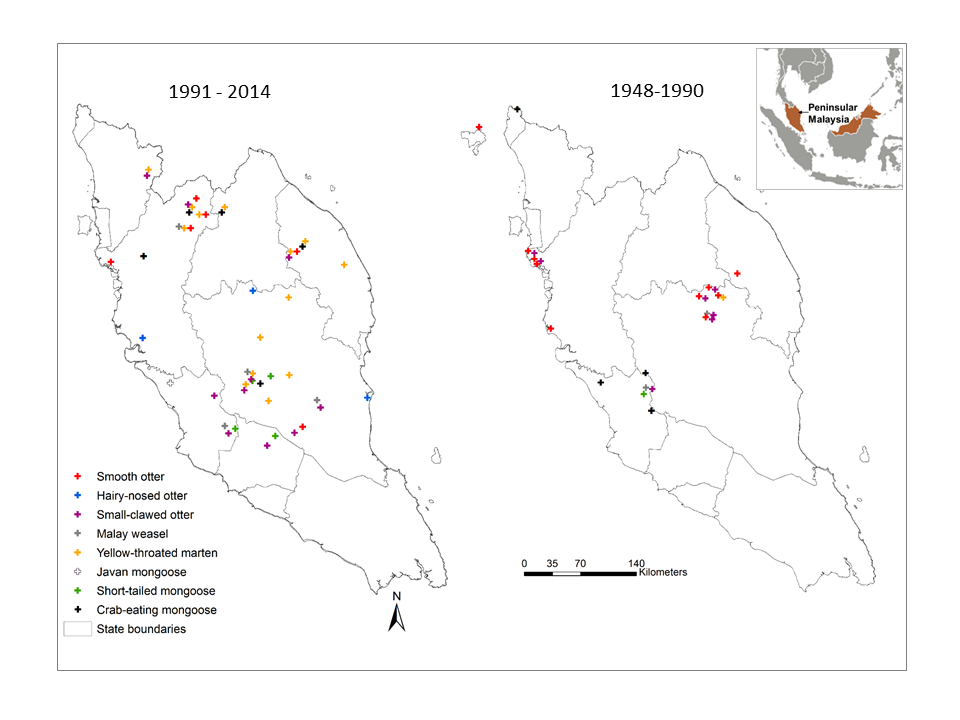
**

**S3 Fig.**

Supplement: S3 Fig — Boundary layer: U.S. State Department, Humanitarian Information Unit (modified from Global Large Scale International Boundary Polygons). Inset map: U.S. Central Intelligence Agency (The World Factbook). (DOCX) [file pone.0194217.s006.docx]

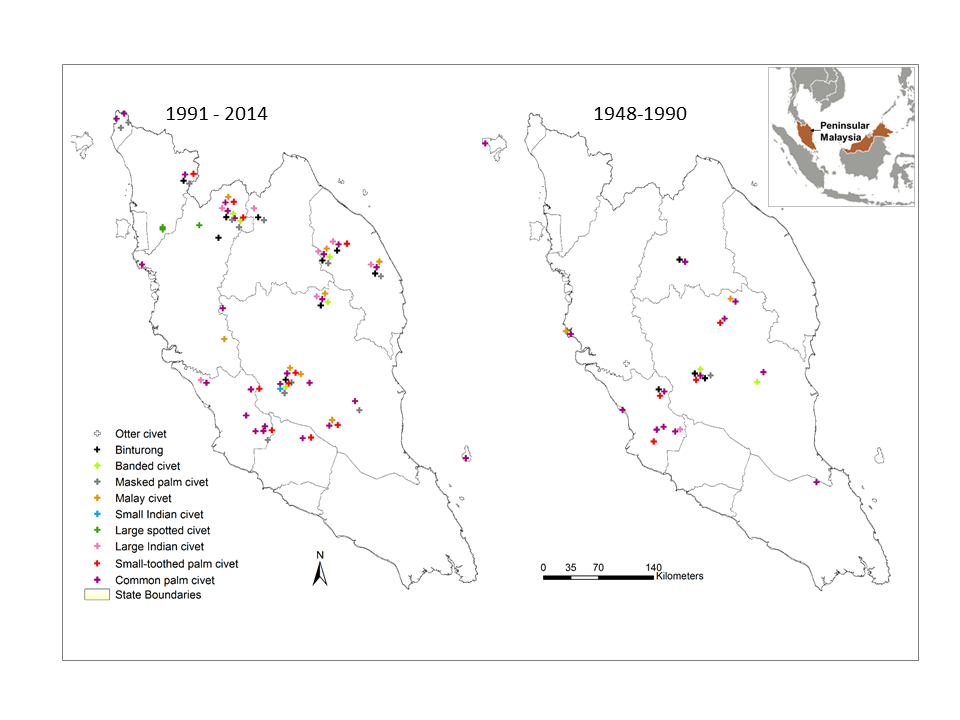


**S4 Fig.**

Supplement: S4 Fig — Boundary layer: U.S. State Department, Humanitarian Information Unit (modified from Global Large Scale International Boundary Polygons). Inset map: U.S. Central Intelligence Agency (The World Factbook). (DOCX) [file pone.0194217.s007.docx]

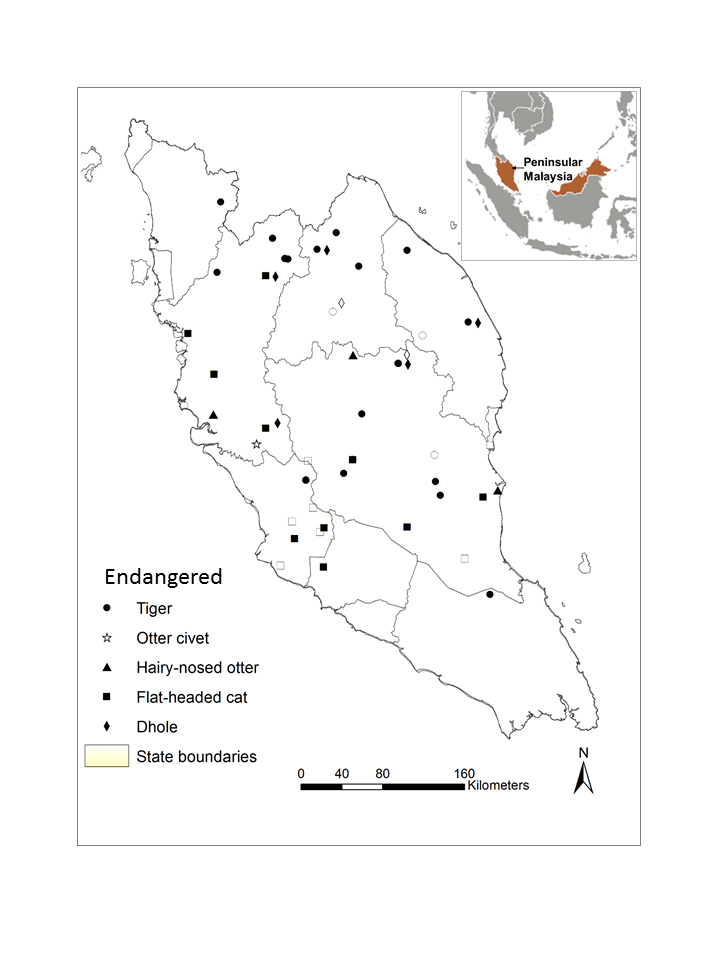


**S5 Fig.**

Supplement: S5 Fig — Solid icons represent recent (1991–2014) records and unfilled icons represent older (1948–1990) records. Boundary layer: U.S. State Department, Humanitarian Information Unit (modified from Global Large Scale International Boundary Polygons). Inset map: U.S. Central Intelligence Agency (The World Factbook). IUCN changed the status of the Malaysian tiger from endangered to critically endangered in 2015. (DOCX) [file pone.0194217.s008.docx]

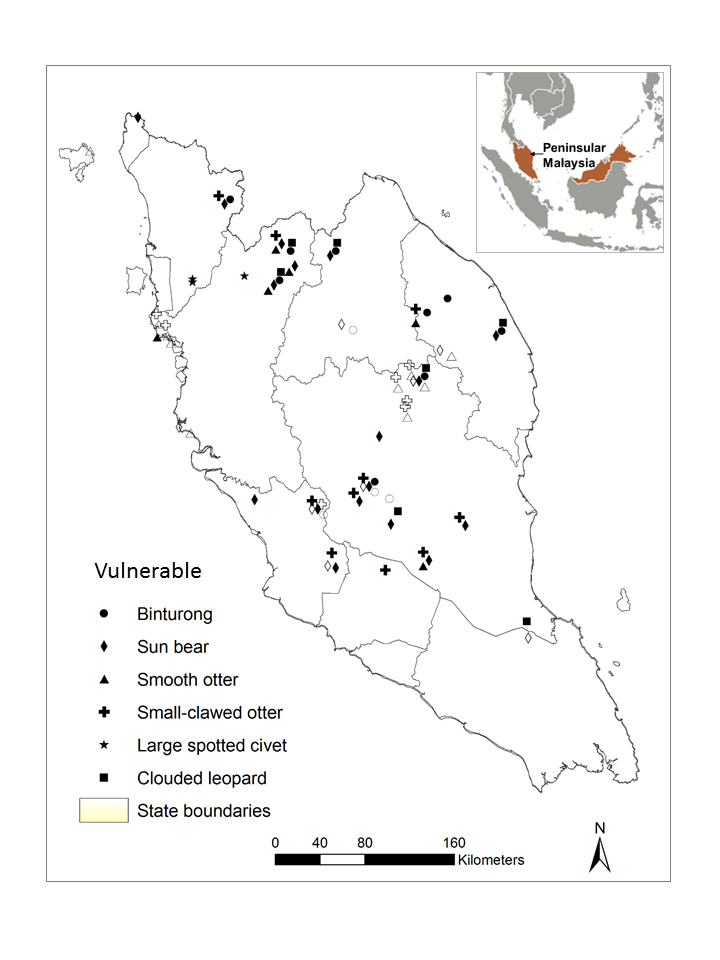


**S6 Fig.**

Supplement: S6 Fig — Solid icons represent recent (1991–2014) records and unfilled icons represent older (1948–1990) records. Boundary layer: U.S. State Department, Humanitarian Information Unit (modified from Global Large Scale International Boundary Polygons). Inset map: U.S. Central Intelligence Agency (The World Factbook). (DOCX) [file pone.0194217.s009.docx]

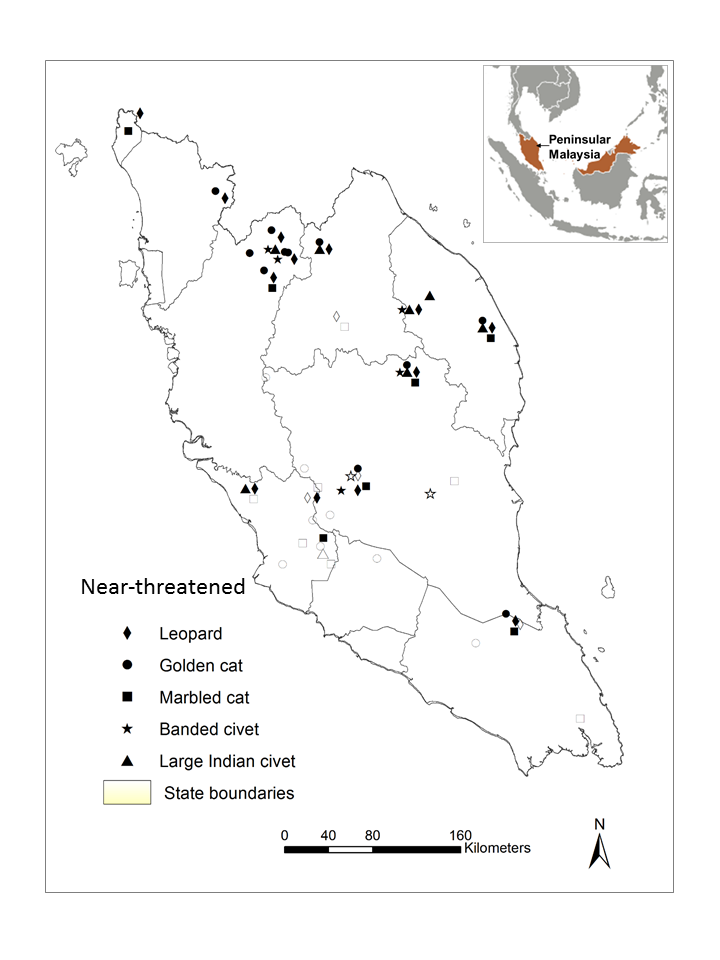


**S7 Fig.**

Supplement: S7 Fig — Solid icons represent recent (1991–2014) records and unfilled icons represent older (1948–1990) records. Boundary layer: U.S. State Department, Humanitarian Information Unit (modified from Global Large Scale International Boundary Polygons). Inset map: U.S. Central Intelligence Agency (The World Factbook). (DOCX) [file pone.0194217.s010.docx]
