# Supplementary material for: Carnivore hotspots in Peninsular Malaysia and their landscape attributes
Source: PLoS One. 2018 Apr 4;13(4):e0194217. doi: 10.1371/journal.pone.0194217 (PMC5884492; doi:10.1371/journal.pone.0194217)
Supplement: S1 Table — (PDF) [file pone.0194217.s011.pdf]

S1 Table. Model selection results to identify landscape variables associated with spatial clustering of carnivore records based on weighted ranking of IUCN red list categories, Peninsular Malaysia, 1948–2014.

**Model no. and variables**[a]	**AIC_c**[b]	**ΔAIC_c**[c]	***K***[d]	**Adjusted model R^2**
1. DCap + RdDens + (DCap × RdDens) + NatHabProp + Elevation	307.64	0	6	0.62
2. DCap + RdDens + (DCap × RdDens) + PopDens + NatHabProp + Elevation	309.22	1.58	7	0.62
3. DCap + RdDens + (DCap × RdDens) + NatHabProp + Elevation + ProxTown	309.80	2.16	7	0.62
4. DCap + RdDens + (DCap × RdDens) + PopDens + Elevation	310.08	2.44	6	0.61
5. DCap + RdDen + (DCapP × RdDens) + NatHabProp	310.19	2.55	5	0.61
6. DCap + RdDens + (DCap × RdDens) + PopDens	310.46	2.82	5	0.62
7. DCap + RdDens + (DCap × RdDens)	310.84	3.20	4	0.61

[a] DCap = distance (m) to capital city (ln transformed; sampling covariate).
RdDens = Density of improved roads (km/km^2) measured within a 15-km radius moving window.
NatHabProp = Proportion of pixels with natural land cover measured within a 15-km radius moving window.
Elevation = elevation (m).
PopDens = human population size per 968- x 968-m grid cell (approximately 1 km^2).
ProxTown = proximity (m) to nearest town or village.
[b] Bias-corrected Akaike's information criterion .
[c] Difference in AIC_c compared with best model.
[d] Number of estimated parameters.
