# Supplementary material for: Carnivore hotspots in Peninsular Malaysia and their landscape attributes
Source: PLoS One. 2018 Apr 4;13(4):e0194217. doi: 10.1371/journal.pone.0194217 (PMC5884492; doi:10.1371/journal.pone.0194217)
Supplement: S2 Table — (PDF) [file pone.0194217.s012.pdf]

S2 Table. Carnivora species reported in Peninsular Malaysia and associated habitats, 1948–2014. Three species considered non-indigenous are excluded.

Species	Mangrove/swamp forest	Lowland forest (<300 m)	Hill forest (300–740 m)	Forests >740 m	Riparian habitat	Oil palm plantations	Orchards	Paddy fields/open scrub	Urban/village	References
Dhole<br>*Cuon alpinus*		✓	✓							Yatim et al. (1985, 1986), Kawanishi et al. (1999), Laidlaw (2000), Azlan and Sharma (2006a), Rayan (2007), Magintan et al. (2010, 2014)
Tiger<br>*Panthera tigris*	✓	✓	✓	✓						Medway (1972), Rayan (2007), (Yatim 1984), Yatim et al. (1985, 1986), Topani (1990), Ratnam et al. (1995), Burhanuddin et al. (1997), Lim et al. (1999a, 2008), Kawanishi et al. (1999), Kassim et al. (1999), Norsham et al. (1999, 2000), Laidlaw (2000), Azlan and Sharma (2006b), Lynam et al. (2007), Ean (2010), Magintan et al. (2010), Gumal et al. (2014), Rayan and Linkie (2015)
Leopard<br>*Panthera pardus*	✓	✓	✓	✓						Medway (1972), Yatim et al. (1986), Sharma (1992), Ratnam et al. (1995), Lim et al. (1999a), Norsham et al. (1999), Kawanishi et al. (1999), Norsham et al. (2000), Azlan and Sharma (2006b), Rayan (2007), Lim et al. (2008), Magintan et al. (2010), Gumal et al. (2014), Sasidhran et al. (2016)
Clouded leopard<br>*Neofelis nebulosa*			✓	✓						Kawanishi et al. (1999), Laidlaw (2000), Azlan and Sharma (2006b), Rayan (2007), Magintan et al. (2010), Gumal et al. (2014), Mohamad et al. (2015)
Marbled cat<br>*Pardofelis marmorata*	✓	✓	✓	✓						Yatim (1983); (Yatim et al. 1986), Kawanishi et al. (1999), Lim (2001), Azlan and Sharma (2006b), Magintan et al. (2010), Gumal et al. (2014)
Leopard cat<br>*Prionailurus bengalensis*	✓	✓	✓		✓	✓	✓	✓	✓	Medway (1972), (Yatim et al. 1986), Burhanuddin et al. (1994), Lim et al. (1995), Ratnam et al. (1995), Lim (1999), Lim et al. (1999a), Lim et al. (1999c), Norsham et al. (1999), Kawanishi et al. (1999), Norsham et al. (2000), Syakirah et al. (2000), Azlan and Sharma (2006b), Rayan (2007), Lim et al. (2008), Lim et al. (2009), Magintan et al. (2010), Gumal et al. (2014)
Flat-headed cat<br>*Prionailurus planiceps*	✓	✓	✓	✓	✓	✓	✓		✓	Lim and Abdul Rahman (1961), Muul and Lim (1970), Muul and Lim (1971), Burhanuddin et al. (1997), Syakirah et al. (2000), Lim and Nazim (2005), Lim et al. (2009), Ean (2010), Magintan et al. (2010)

Asian golden cat<br>*Catopuma temminckii*	✓	✓	✓	✓						Kawanishi et al. (1999), Norsham et al. (2000), Lim (2002), Azlan and Sharma (2006b), Rayan (2007), Magintan et al. (2010), Gumal et al. (2014),
Javan mongoose<br>*Herpestes javanicus*	✓							✓		Wells (1989), Sasidhran et al. (2016)
Short-tailed mongoose<br>*Herpestes brachyurus*		✓	✓		✓	✓				Lim et al. (1995), Lim et al. (1999c), Lim et al. (2008), Lim et al. (2009) , Jennings et al. (2010a)
Crab-eating mongoose<br>*Herpestes urva*	✓	✓	✓	✓	✓					Wells and Francis (1988), Saharudin (1990), Rayan (2007), Rayan and Shariff (2008), Hedges et al. (2013), Ean et al. (2014)
Yellow-throated marten<br>*Martes flavigula*		✓	✓							Yatim et al. (1986), Norsham et al. (1999), Kawanishi et al. (1999), Laidlaw (2000), Azlan and Sharma (2006a), Rayan (2007), Lim et al. (2008), Magintan et al. (2010), Hedges et al. (2013)
Malay weasel<br>*Mustela nudipes*	✓	✓			✓					(Lim et al. 1989), Lim et al. (1999a), Lim et al. (1999c), Lim et al. (2009), Magintan et al. (2010)
Asian small-clawed otter<br>*Aonyx cinerea*	✓	✓	✓		✓			✓		(Shariff 1984), Shariff (1985), Lim et al. (1989), Burhanuddin (1989), Burhanuddin and Norizan (1990), Foster-Turley (1992), Lim et al. (1995), Lim et al. (1999a), Lim et al. (1999c), Norsham et al. (1999), Norsham et al. (2000), Syakirah et al. (2000), Lim et al. (2008), Lim et al. (2009), Hedges et al. (2013)
Hairy-nosed otter<br>*Lutra sumatrana*	✓	✓			✓					Sebastian (1995), Baker (2013)
Smooth otter<br>*Lutrogale perspicillata*	✓	✓	✓		✓	✓		✓		Yatim (1984), Shariff (1984), Shariff (1985), Yatim et al. (1985), Burhanuddin (1989), Burhanuddin and Norizan (1990), Foster-Turley (1992), Burhanuddin et al. (1994), Ratnam et al. (1995), Norsham et al. (2000), Syakirah et al. (2000), Magintan et al. (2010), Hedges et al. (2013)
Banded linsang<br>*Prionodon linsang*		✓	✓	✓	✓					Medway (1972), Lim (1973), Lim et al. (1995), Ratnam et al. (1995), Lim et al. (1999c), Norsham et al. (1999), Azlan and Sharma (2006a), Rayan (2007), Lim et al. (2009), Magintan et al. (2010), Hedges et al. (2013), Jennings and Veron (2014)
Malayan sun bear<br>*Helarctos malayanus*	✓	✓	✓	✓		✓				Medway (1972), Yatim (1983), Yatim (1984), Yatim et al. (1985); (Yatim et al. 1986), Burhanuddin et al. (1997), Sharma (1992), Ratnam et al. (1995), Kassim et al. (1999), Lim et al. (1999a), (Lim et al. 1999c), Norsham et al. (1999), Laidlaw (2000), Norsham et al. (2000), Norsham and Ong (2001), Rayan (2007), Magintan et al. (2010), Ean (2010)
Small Indian civet<br>*Viverricula indica*		✓								Lim et al. (2008)
Malay civet<br>*Viverra tangalunga*		✓	✓							Yatim (1984), Yatim et al. (1985), Kawanishi et al. (1999), Norsham et al. (2000), Syakirah et al. (2000), Azlan and Sharma (2006a), Jennings et al. (2010b), Jennings and

										Veron (2011), Hedges et al. (2013)
Large spotted civet<br>*Viverra megaspila*						✓				Hamirul et al. (2015)
Large Indian civet<br>*Viverra zibetha*	✓	✓	✓							Yatim (1983), Azlan and Sharma (2006a), Rayan (2007), Jennings and Veron (2011); Hedges et al. (2013), Kawanishi et al. (1999), Mohamad et al. (2015)
Otter civet<br>*Cyanogale bennetti*	✓									Abdul (1987)
Masked palm civet<br>*Paguma larvata*		✓	✓							Lambert (1990), Sharma (1992), Ratnam et al. (1995), Norsham et al. (1999), Lim et al. (1999a), Norsham and Ong (2001), Azlan and Sharma (2006a), Rayan (2007), Lim et al. (2008), Hedges et al. (2013), Jayaraj et al. (2013)
Common palm civet<br>*Paradoxurus hermaphroditus*	✓	✓	✓	✓	✓	✓	✓		✓	Medway (1972), Yatim (1983, 1984), Yatim et al. (1985, 1986), Lim et al. (1989), Sharma (1992), Shariff and Hamid (1993), Burhanuddin et al. (1994, 1997), Lim et al. (1995), Ratnam et al. (1995), Lim et al. (1999a,b,c, 2008, 2009), Norsham et al. (1999, 2000), Kawanishi et al. (1999), Azlan and Sharma (2006a), Ean (2010), Jayaraj et al. (2013), Hedges et al. (2013)
Banded civet<br>*Hemigalus derbyanus*		✓	✓		✓					Medway (1972), Lim (1973), Ratnam et al. (1995), Kawanishi et al. (1999), Lim et al. (2008), Hedges et al. (2013)
Small-toothed palm civet<br>*Arctogalidia trivirgata*	✓	✓	✓		✓		✓			Medway (1972), (Ratnam 1987), Hedges et al. (2013), (Lim et al. 1989), Lim et al. (1995), Ratnam et al. (1995), Lim et al. (1999a), Norsham et al. (1999), Norsham et al. (2000), Syakirah et al. (2000), Lim et al. (2008), Lim et al. (2009)
Binturong<br>*Arctitis binturong*		✓	✓							Medway (1972), (Yatim et al. 1986), Lim et al. (1999a), Norsham et al. (1999), Kawanishi et al. (1999), Azlan and Sharma (2006a), Rayan (2007), Magintan et al. (2010), Hedges et al. (2013)
